# The Structure and Properties of Polyacrylonitrile Nascent Composite Fibers with Grafted Multi Walled Carbon Nanotubes Prepared by Wet Spinning Method

**DOI:** 10.3390/polym11030422

**Published:** 2019-03-05

**Authors:** Hailong Zhang, Ling Quan, Aijun Gao, Yuping Tong, Fengjun Shi, Lianghua Xu

**Affiliations:** 1 School of Materials Science and Engineering, North China University of Water Resources and Electric Power, Zhengzhou 450045, China; tongyuping@ncwu.edu.cn (Y.T.); shifengjun1962@126.com (F.S.); 2 School of Electric Power, North China University of Water Resources and Electric Power, Zhengzhou 450045, China; quanling@ncwu.edu.cn; 3 Key Laboratory of Carbon Fiber and Functional Polymers Ministry of Education, Beijing University of Chemical Technology, Beijing 100029, China; bhgaoaijun@163.com (A.G.); xulh@mail.buct.edu.cn (L.X.)

**Keywords:** multi walled carbon nanotubes, polyacrylonitrile, nascent fiber, thermal properties, morphological structure

## Abstract

Polyacrylonitrile (PAN) grafted amino-functionalized multi walled carbon nanotubes (amino-MWCNTs) were synthesized by in situ polymerization under aqueous solvent. The grafted MWCNT/PAN nascent composite fibers were prepared by the wet spinning method. Fourier transform infrared spectroscopy and Raman spectroscopy indicated that the amino-MWCNTs and PAN macromolecular chains had interfacial interactions and formed chemical bonds. The grafting content of the PAN polymer on the amino-MWCNTs was up to 73.2% by thermo gravimetric analysis. The incorporation of the grafted MWCNTs improved the degree of crystallization and crystal size of PAN nascent fibers, and changed the thermal properties during exothermic processing in an air atmosphere. Morphology analysis and testing of mechanical properties showed that the grafted MWCNT/PAN nascent composite fibers with a more uniform diameter distribution and larger diameter had higher tensile strength and tensile modulus than the control PAN nascent fibers.

## 1. Introduction

Owing to their unique structure and properties, such as high strength, low mass density, and large aspect ratio, carbon nanotubes (CNTs) have been used as an ideal reinforcing agent in composites [1,2]. However, CNTs are easily aggregated because of a strong van der Waals force between them and large specific surface area, which makes CNTs difficult to disperse into most solvents or polymer matrices. Moreover, it is difficult for strong interfacial interactions to form between the inert surface of the multi wall carbon nanotubes (MWCNTs) and the polymer matrix, which limits the excellent properties of the MWCNTs. Thus, various non-covalent and covalent methods are used to modify the surface of CNTs for improving the dispersion and interfacial interactions. The non-covalent method is based on the intermolecular interaction on the surface of CNTs, physical adsorption and/or wrapping [3], but the weak interfacial interaction between the CNTs and polymer matrix limits the effective transfer of stress. The covalent method is based on the formation of a chemical bond on the outer CNT wall, which can form a strong interfacial interaction between CNTs and the functionalized agent [4,5]. Katti et al. [6] reported that the poly(ether ether ketone) grafted on carboxyl functionalized MWCNTs improved the dispersion of MWCNTs in the epoxy resin by mechanical stirring and exhibited significant enhancement in mechanical properties. Konnola et al. [7] showed that hydroxyl terminated poly(ether sulfone) grafted on multi walled carbon nanotubes obviously increased the tensile strength and fracture toughness of the epoxy matrix. Therefore, the covalent method is considered, by many authors, an effective method to modify CNTs in order to obtain CNT/polymer matrix composites [8,9,10,11]. 

Polyacrylonitrile (PAN) is an important precursor for preparing high performance carbon fibers. The structure and properties of nascent fibers prepared by wet spinning are formed by liquid solid phase separation under stretching, which influences the evolution of subsequent properties during the preparation of PAN precursor fibers, and affects the final properties of carbon fibers. Up to now, CNT/PAN composite precursor fibers have been reported by many researchers [12,13,14,15,16], but the CNTs were often directly dispersed in the organic solvent and needed to be removed in the last processing, which polluted the environment. However, in situ polymerization provides an effective way to solve this problem, and could improve the interfacial interaction and dispersion of the CNTs. Zhou et al. [17] reported that multi walled carbon nanotube/polyacrylonitrile composite fibers were obtained by in situ polymerization, which exhibited better dispersion of CNTs and interfacial interaction between PAN and CNTs compared with fibers prepared by mechanical mixing. Poochai et al. [18] reported that PAN grafted CNTs by admicellar polymerization would improve the dispersion of CNTs in the PAN matrix. However, the effects of grafted CNTs on the structure and mechanical properties for the PAN nascent fibers were rarely reported. Although our research teams have reported the orientation, crystal structure and thermal properties of CNT/PAN nascent composite fibers [19], grafted CNT/PAN nascent composite fibers prepared by the wet spinning method need to be further researched and discussed.

In this paper, PAN grafted amino-functionalization MWCNT (amino-MWCNT) nanocomposites were synthesized by in situ polymerization under aqueous solvent with ultra-sonication. The nanocomposites, acrylonitrile (AN) and itaconic acid (IA) monomer, were dissolved in dimethylsulphoxide (DMSO) and initiated by solution polymerization to obtain the composite spinning solutions. The grafted MWCNT/PAN nascent composite fibers were prepared by the wet spinning method. The interfacial interaction between amino-MWCNTs and PAN polymers was characterized by Fourier transform infrared (FTIR) spectroscopy and Raman spectroscopy. The grafting amount of PAN polymer was performed by thermo gravimetric (TG) analysis. The effects of the grafted MWCNTs on the crystal structure, thermal properties, cross-sectional structure and surface morphology, as well as mechanical properties of the PAN nascent fibers were analyzed by X-ray diffraction (XRD), differential scanning calorimetry (DSC), transmission electron microscopy (TEM), scanning electron microscopy (SEM), and a monofilament tensile testing machine, respectively.

## 2. Experiment

### 2.1. Materials

The pristine MWCNTs (≥95 wt %) with 10–15 nm diameter and 1–10 μm length were purchased from Shenzhen Nanotech Port Co., Ltd. (Shenzhen, China), synthesized using the chemical vapor deposition method. Acrylonitrile (AN) was provided by Beijing Xingjin Chemical Factory (Beijing, China) and purified before polymerization. DMSO and IA were supplied by the Beijing Yili Reagent Corporation (Beijing, China). Hydrochloric acid (HCl), hydrogen peroxide (H_2_O_2_), thionyl chloride (SOCl_2_), tetrahydrofuran (THF) and triethylenetetramine (TETA) were obtained from Sinopharm Chemical Reagent Co., Ltd. (Shanghai, China).

### 2.2. Preparation of the Amino-MWCNTs

**Figure 1:**
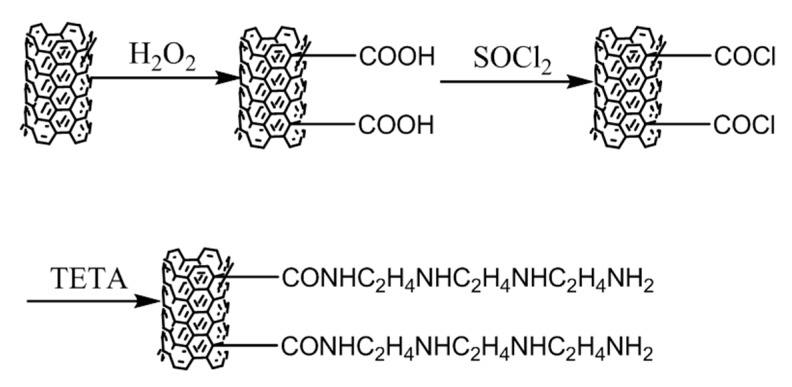
Schematic representation of the formation of amino-MWCNTs.

The pristine MWCNTs were first purified by HCl to remove the metallic catalysts. The purified MWCNTs were dispersed in H_2_O_2_ solution with a concentration of 30% under ultrasonic bath for 2 h, and then stirred at 70 °C for 24 h to obtain the MWCNTs with a carboxyl group. The obtained MWCNTs were refluxed with SOCl_2_ solution under ultra-sonication at 23 °C for 2 h and stirred at 75 °C for 24 h. The MWCNTs with a Cl element were reacted with excessive TETA by ultrasound for 2 h and stirred at 120 °C for 24 h to obtain the amino-functionalized MWCNTs (amino-MWCNTs). The schematic route is shown in Figure 1.

### 2.3. PAN Grafted Amino-MWCNT Nanocomposites

The PAN grafted amino-MWCNT nanocomposites were synthesized in aqueous solvent by in situ polymerization under ultra-sonication. The AN monomer was dispersed in aqueous solution by ultrasonic treatment for 10 min under nitrogen atmosphere. The certain amounts of amino-MWCNTs were placed into the mixed solution by ultrasound at 50 °C for 30 min. The initiator was added and the mixture solution begun to polymerize for 2 h. Finally, the mixture was filtered and dried under vacuum at 40 °C. The control PAN polymer was synthesized under the same conditions without amino-MWCNTs.

### 2.4. The Grafted MWCNT/PAN Nascent Composite Fibers

The nanocomposites were dissolved in DMSO solvent under an ultra-sonication bath in a nitrogen atmosphere for 5 min. The AN and IA monomers were added into solvent, and then initiated by the initiator after 10 min to obtain the grafted MWCNT/PAN composite spinning solution with a concentration of about 22%. The grafted MWCNT/PAN nascent composite fibers were prepared by the wet spinning method. The mass concentration of MWCNTs in the nascent composite fibers was about 1%. The control PAN nascent fibers were prepared under the same conditions without the grafted MWCNTs.

### 2.5. Characterization

The chemical bonds were characterized by FTIR spectroscopy (Nicolet 5700 spectrometer, Thermo Nicolet Company, Madison, WI, USA), and the samples were pressed into a pellet with potassium bromide (KBr). The spectra obtained ranged from 400 to 4000 cm^−1^ at a resolution of 4 cm^−1^. The Raman spectrum was recorded using a Microscopic Confocal Raman Spectrometer (RM2000, Renishaw, London, UK) with a He-Ne laser (Spectra-Physics) excitation at 632.8 nm. The amino-MWCNTs and the PAN grafted amino-MWCNTs were deposited on glass slides, and the laser beam was focused with a 5 μm diameter spot on the samples. Morphologies of amino-MWCNTs and the PAN grafted amino-MWCNT nanocomposites were directly observed by transmission electron microscopy (H-800, Hitachi, Tokyo, Japan) at an operating voltage of 200 kV. The samples were dispersed into alcohol solvent under ultrasonic bath at 40 kHz for 5 min, and then dripped onto the copper grid covered with a perforated carbon film. The cross-sectional structure and surface morphology of the PAN nascent fibers and the grafted MWCNT/PAN nascent composite fibers were examined by SEM (S 4700, Hitachi, Tokyo, Japan) at an operating voltage of 20 kV. The nascent fibers were cured in epoxy resin, and then fractured in liquid nitrogen to observe the cross-sectional structure after sputtering gold. The samples were fixed on the surface of a sample table and sputtered with gold to examine the surface morphology. The diameter distribution was carried out using ImageJ software (National Institutes of Health, Berhesda, Rockville, MD, USA), and 20 positions in a SEM image were chosen for analyzing. The thermal stabilities of the PAN polymer and the different MWCNTs were evaluated using a thermo gravimetric instrument (TG 209, Netzsch, Bavarian State, Selb, Germany). Specimens were heated from 50 to 800 °C at a heating rate of 20 °C/min in a nitrogen atmosphere. DSC was carried out in an air atmosphere using a Q 100 instrument (TA Company, Boston, PA, USA). Samples of about 3.0 mg were used to evaluate the exothermic properties of the different nascent fibers at 10 °C heating rate from 50 to 400 °C. The mechanical properties were measured by a monofilament tensile machine (YG001N, Nantong Hongda Company, Nantong, Jiangsu, China). The specimens were tested at a crosshead speed of 5 mm/min with gage length of 50 mm at room temperature. The number of tensile tests performed was 25. XRD was recorded using a D/max 2500VB2+/PC diffractometer (Rigaku, Japan) with a range of 2θ from 5° to 55° at the scanning speed of 10°/min and a step of 1.0 s. The operation was used at 40 kV and 50 mA with Cu Kα radiation (wavelength λ = 0.154056 nm). The degree of crystallization and the crystal size were calculated according to the formula $C(\%)=A_{c}/(A_{a}+A_{c})\times 100\%$ and the Scherrer equation $L_{c}=K\lambda /(\beta \cos\theta )$, respectively. Where *C* % is the degree of crystallization, *A_c_* is the area of the crystalline region, *A_a_* is the area of the amorphous region; *L_c_* is the crystal size, *K* is 0.89, λ = 0.154056 nm is the wavelength of the XRD, and β is the full width at half maximum at 2θ ≈ 17° [20,21].

## 3. Results and Discussion

**Figure 2:**
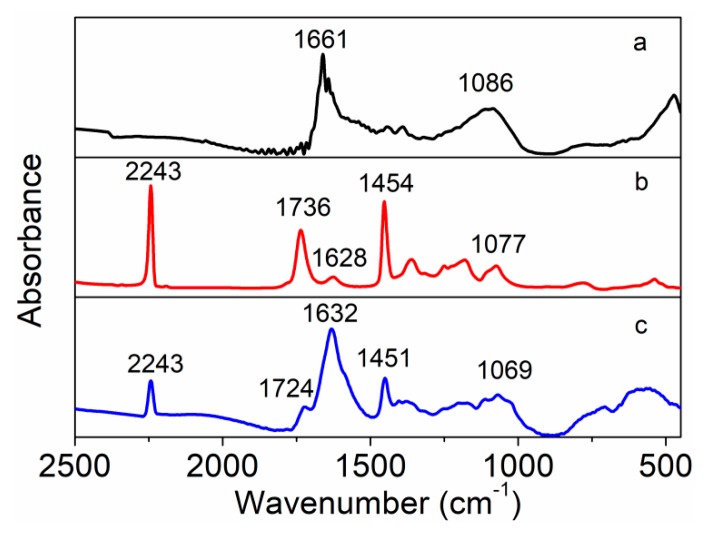
FTIR spectra of (**a**) amino-MWCNTs, (**b**) PAN polymer and (**c**) PAN grafted amino-MWCNT nanocomposites.

The FTIR spectra of amino-MWCNTs, PAN polymer and PAN grafted amino-MWCNT nanocomposites are shown in Figure 2. The FTIR spectrum of amino-MWCNTs, as can be seen in Figure 2a, showed a very strong absorption peak at 1661 cm^−1^, which is assigned to the stretching vibration of amide carbonyl groups, and the band at 1086 cm^−1^ is attributed to the stretching vibration of the C–N bond. These results are consistent with previous reports [22,23], indicating that the amino groups are grafted onto the surface of MWCNTs. The FTIR spectrum of the control PAN polymer showed a very strong absorption peak at about 2243 cm^−1^, which is attributed to the nitrile group vibration, and the peak at 1736 cm^−1^ corresponds to the carboxylic acid groups of IA on the PAN macromolecular chains [24]. The peaks at 1454 and 1077 cm^−1^ are assigned to the bending vibration of C–H groups. The weak peak at 1628 cm^−1^ belongs to the NH_2_ bending that was likely caused by the hydrolysis of nitrile groups [25]. For the PAN grafted amino-MWCNT nanocomposites, the peak of the carboxylic acid group shifted from 1736 to 1724 cm^−1^, and the peak of C–H bending vibration shifted from 1454 to 1451 cm^−1^ and from 1077 to 1069 cm^−1^, respectively. Moreover, the strong peak of amino-MWCNTs shifted from 1661 to 1632 cm^−1^ of the PAN grafted amino-MWCNT nanocomposites. The results coming from these shifted peaks suggest that the amino-functionalized groups on the surface of MWCNTs reacted with the carboxylic acid groups on the control PAN macromolecule chains, and a chemical bond was formed between them.

**Figure 3:**
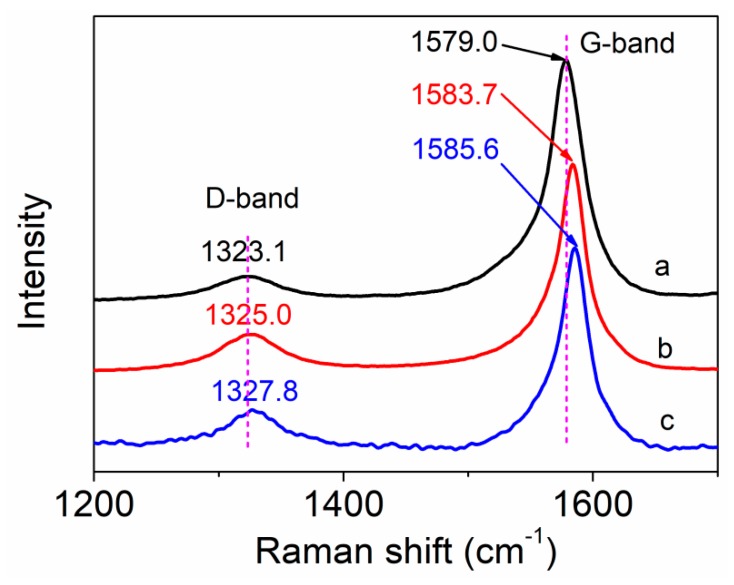
Raman spectra of (**a**) purified MWCNTs, (**b**) amino-MWCNTs and (**c**) PAN grafted amino-MWCNT nanocomposites.

Raman spectroscopy was used to characterize the structural changes of the surface of MWCNTs, and the interfacial interaction between the MWCNTs and polymer matrix according to the shift of peaks about MWCNTs. The purified MWCNTs, as shown in Figure 3a, had two significant peaks. The strong peak at about 1579.0 cm^−1^ (G-band) is assigned to the sp^2^ graphite carbon of the MWCNTs, and the broad weak peak at about 1323.1 cm^−1^ (D-band) is attributed to the sp^3^ hybridized carbon of the MWCNTs [26]. After being modified by amino-functionalized groups, as shown in Figure 3b, the amino-MWCNTs also had two typical peaks. However, the peaks about the G-band and D-band shifted from 1579.0 to 1583.7 cm^−1^ and from 1323.1 to 1325.0 cm^−1^, respectively. Compared with the amino-MWCNTs, the peak of the G-band for the PAN grafted amino-MWCNT nanocomposites shifted from 1583.7 to 1585.6 cm^−1^, and the peak of the D-band shifted from 1325.0 to 1327.8 cm^−1^. These results indicate that a strong chemical interfacial interaction exists between the PAN and the amino-MWCNTs after in situ polymerization [27]. The value of the G-band/D-band intensity ratio (I_G_/I_D_) was used to characterize the surface structure of MWCNTs. The value of I_G_/I_D_ for amino-MWCNTs is 5.98, which is lower than that of purified MWCNTs at 9.84. This suggests that the surface of the amino-MWCNTs has more relative content of sp^3^ hybridized carbon than the purified MWCNTs owing to grafting of the amino-functionalized groups. However, the value of I_G_/I_D_ for the PAN grafted amino-MWCNT nanocomposites is 4.87, indicating that the relative number of sp^3^ hybridized carbon attaching onto the amino-MWCNTs increased after AN polymerization [28]. Thus, the Raman results suggest that the PAN polymer covalently attached to the surface of the amino-MWCNTs.

**Figure 4:**
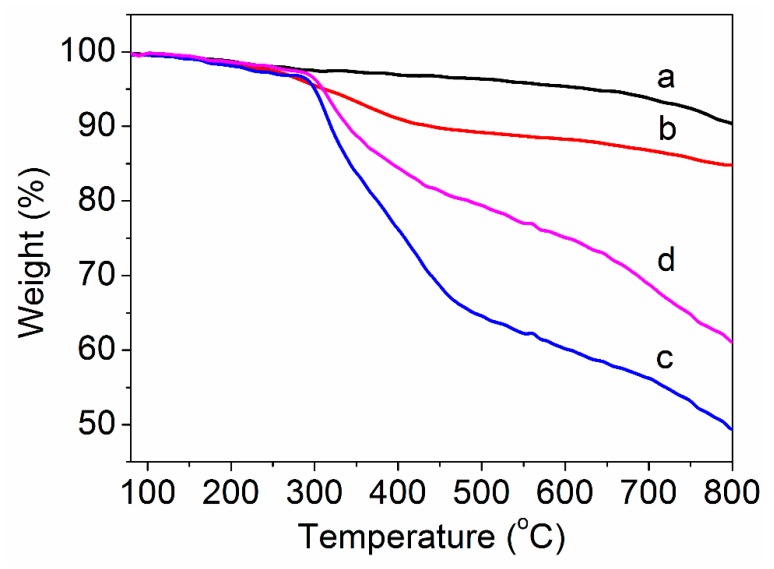
TG curves of (**a**) purified MWCNTs, (**b**) amino-MWCNTs, (**c**) PAN polymer and (**d**) PAN grafted amino-MWCNT nanocomposites.

The relative amount of the grafted PAN can be calculated by TG curves through thermal decomposition. The TG curves of purified MWCNTs, amino-MWCNTs, the PAN polymer and the PAN grafted amino-MWCNT nanocomposites in a nitrogen atmosphere are shown in Figure 4. As can be seen in Figure 4a, the weight loss of purified MWCNTs was 9.6% at 800 °C. For the amino-MWCNTs, as shown in Figure 4b, rapid weight loss occurred between 250 and 400 °C, which is ascribed to some functional groups on the surface of MWCNTs. The weight loss of the amino-MWCNTs at 800 °C was 15.2%, which is larger than that of purified MWCNTs. The PAN polymer had similar weight loss to PAN grafted amino-MWCNTs at lower temperatures between 80 and 300 °C, as shown in Figure 4c,d, which is attributed to the loss of the residual water and small molecules. At a temperature above 300 °C, the PAN polymer begun to lose weight quickly up to 470 °C, and then the rate of weight loss slowed down. During this process, the PAN polymer underwent a cyclized reaction, dehydrogenation reaction and oxidative reaction to form a ring structure. The weight loss for the PAN polymer at 800 °C was 50.5%. However, the rate of weight loss for the PAN grafted amino-MWCNTs was lower than that of the PAN polymer for a temperature from 300 to 470 °C, and had the same parallel line as the PAN polymer between 470 and 800 °C. The weight loss of the PAN grafted amino-MWCNTs was 38.9% at 800 °C. The higher residual weight suggests that the amino-MNCNTs changed the complex chemical reaction and promoted formation of a more stable ring structure because of the interfacial interaction between them [29], which was beneficial for obtaining a high yield of the carbon materials. According to the weight loss at 800 °C for the amino-MWCNTs, PAN polymer and PAN grafted amino-MWCNTs, the content of grafted PAN on the surface of amino-MWCNTs was about 73.2%.

**Figure 5:**
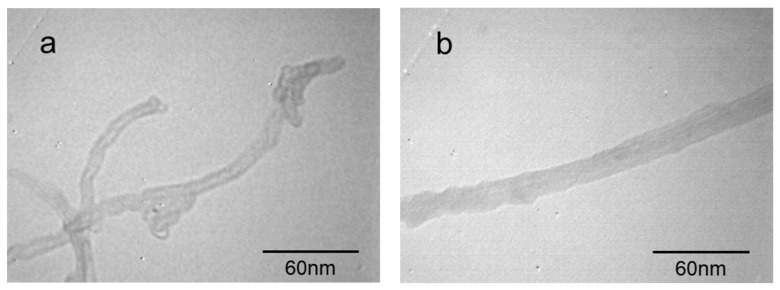
TEM images of (**a**) amino-MWCNTs and (**b**) PAN grafted amino-MWCNTs nanocomposites.

To confirm the PAN polymer grafted onto the surface of the amino-MWCNTs, the TEM images of amino-MWCNTs and the PAN grafted amino-MWCNTs nanocomposites are shown in Figure 5. The amino-MWCNTs, having a smooth surface, are individually dispersed with a diameter of about 10 nm, as shown in Figure 5a, and have a hollow structure with an internal diameter of about 5 nm. However, the PAN grafted amino-MWCNTs, as can be seen in Figure 5b, exhibit a non-hollow structure owing to the grafted PAN polymer layer, with a diameter of about 20–25 nm and a rough surface. These results suggest that the PAN polymer was grafted onto the surface of the amino-MWCNTs by in situ polymerization.

**Figure 6:**
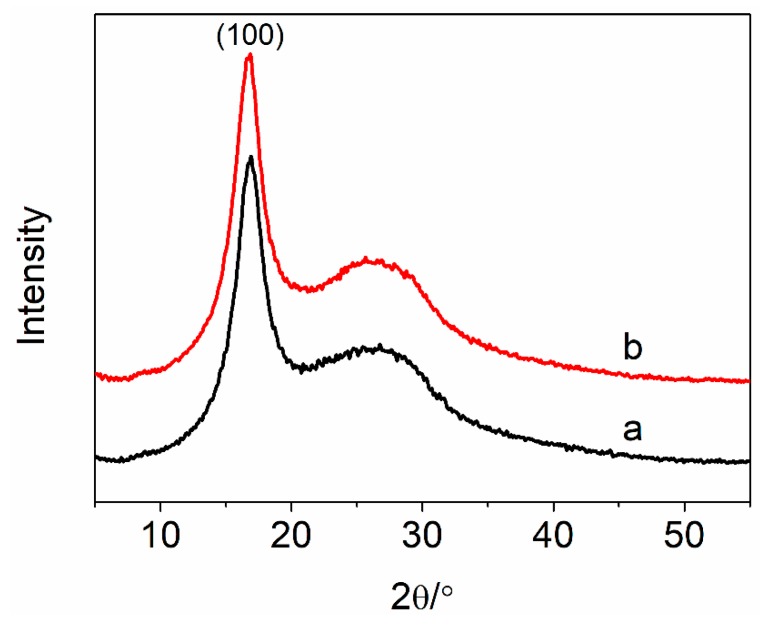
XRD curves of (**a**) the PAN nascent fibers and (**b**) the grafted MWCNT/PAN nascent composite fibers.

XRD curves of the PAN nascent fibers and the grafted MWCNT/PAN nascent composite fibers are shown in Figure 6. A PAN macromolecule is considered as a semi-crystalline polymer because of the strong interaction of the nitrile groups. PAN nascent fibers show a strong absorption peak at around 16.8°, which is ascribed to the (100) crystal plane. The weak peak at about 25.6° is assigned to the amorphous region [30]. Because the mass concentration of the grafted MWCNTs in the composite fibers is small, the grafted MWCNT/PAN nascent composite fibers show a similar shape to the PAN nascent fibers, indicating that the incorporation of grafted MWCNTs would not change the crystal structure of PAN nascent fibers. From the XRD curves, the degree of crystallization and the crystal size of different nascent fibers are listed in Table 1. 

From Table 1, it can be seen that the degree of crystallization of the PAN nascent fibers was 35.23%, which was calculated from the (100) crystal plane. The crystal size of the PAN nascent fibers was 3.01 nm, which is assigned to the rod-like structure due to the intermolecular repulsion of the nitrile dipoles [31]. The degree of crystallization of the grafted MWCNT/PAN nascent composite fibers was 38.73%, which is higher than that of the PAN nascent fibers. Meanwhile, the addition of grafted MWCNTs into the PAN nascent fibers improved the crystal size from 3.01 to 3.42 nm. This is attributed to the MWCNTs with a small diameter as the nucleating agent, which could induce growth of PAN crystallites, and transform the amorphous regions around the MWCNTs into a crystal region, resulting in an improvement in the degree of crystallization [32].

**Figure 7:**
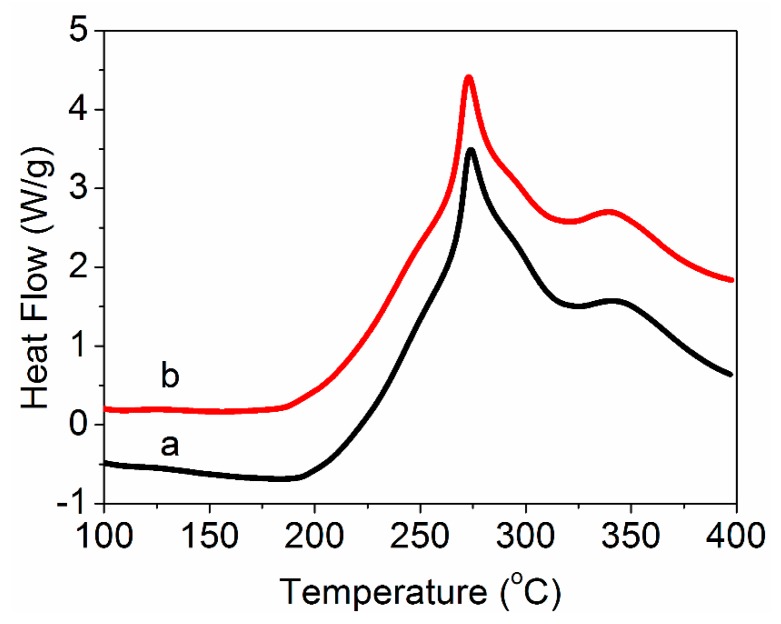
DSC curves of (**a**) the PAN nascent fibers and (**b**) the grafted MWCNT/PAN nascent composite fibers.

In order to investigate the effect of grafted MWCNTs on the thermal properties of PAN nascent fibers during the thermal stabilization, the DSC curves of the PAN nascent fibers and grafted MWCNT/PAN nascent composite fibers under an air atmosphere are shown in Figure 7. It was clearly seen that the DSC curves displayed two exothermic peaks. The first strong peak is attributed to the cyclization reaction of the PAN polymer, and the second weak peak is assigned to the oxygen reaction. The initial temperature (*T*_i_), the first peak temperature (*T*_1_), the second temperature (*T*_2_), the finish temperature (*T*_f_) and the evolved heat (Δ*H*) are shown in Table 2. 

According to the data in Table 2, the grafted MWCNT/PAN nascent composite fibers have a much lower value of *T*_i_ than the PAN nascent fibers, indicating a greater ease of initiation of stabilization in an air atmosphere. This phenomenon may be ascribed to the grafted MWCNTs inducing the onset temperature of the cyclization reaction. The grafted MWCNT/PAN nascent composite fibers had a lower *T*_1_, *T*_2_ and *T*_f_ than the PAN nascent fibers, which could be assigned to the MWCNTs having high heat conductivity. However, the grafted MWCNT/PAN nascent composite fibers with a high degree of crystallization had lower ΔH and broader ΔT than the PAN nascent fibers, suggesting that the nascent composite fibers were more easily controlled during the thermal stabilization process. 

**Figure 8:**
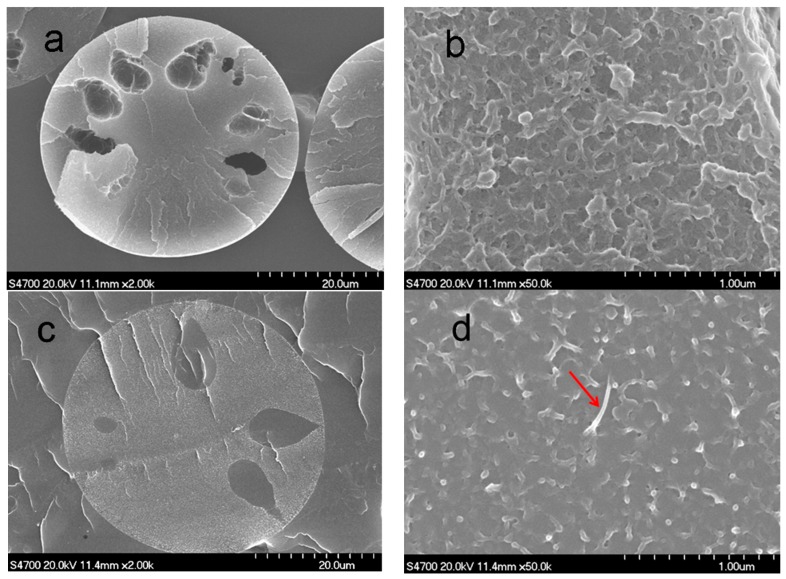
SEM images of cross sectional structure: (**a**,**b**) the PAN nascent fibers and (**c**,**d**) the grafted MWCNT/PAN nascent composite fibers.

Figure 8 shows the cross-sectional structure of the PAN nascent fibers and the grafted MWCNT/PAN nascent composite fibers. The cross-sectional structure of the PAN nascent fibers in Figure 8a shows some porous structures between the skin and core, which is attributed to the exchange of water and DMSO solvent when the spinning solution passes through the spinneret holes and spins into the coagulation bath. Furthermore, there are some nanopores on the cross-sectional morphology in Figure 8b. However, the addition of grafted MWCNTs into the PAN matrix reduces the number of porous structures and nanopores in the grafted MWCNT/PAN composite fibers, as shown in Figure 8c,d. Especially as shown by the arrow in Figure 8d, the individual grafted MWCNTs with a diameter about 20 nm was exhibited in the cross-section of the nascent composite fibers. Moreover, the morphology of cross-section for the PAN nascent fibers shows a lamellar structure in Figure 8b. However, the composite fibers in Figure 8d exhibit some wire drawing on the cross-section owing to the PAN macromolecular chains. These structures indicate that the grafted MWCNT/PAN nascent composite fibers have better tensile properties than the PAN nascent fibers. Meanwhile, a less porous structure means higher compactness, and higher compactness suggests that the nascent fibers have higher mechanical properties, as shown in Table 3. The addition of the grafted MWCNTs increases the tensile strength from 3.83 to 4.37 cN/dtex and the tensile modulus from 101.73 to 121.82 cN/dtex, and decreases the breaking elongation from 50.63% to 32.68%, respectively. Moreover, this porous structure would continue to be retained in the PAN precursor fibers through the drawing process, which reduced the tensile strength of the final carbon fibers. Therefore, the grafted MWCNT/PAN nascent composite fibers would achieve high performance carbon fibers compared with the PAN nascent fibers. 

**Figure 9:**
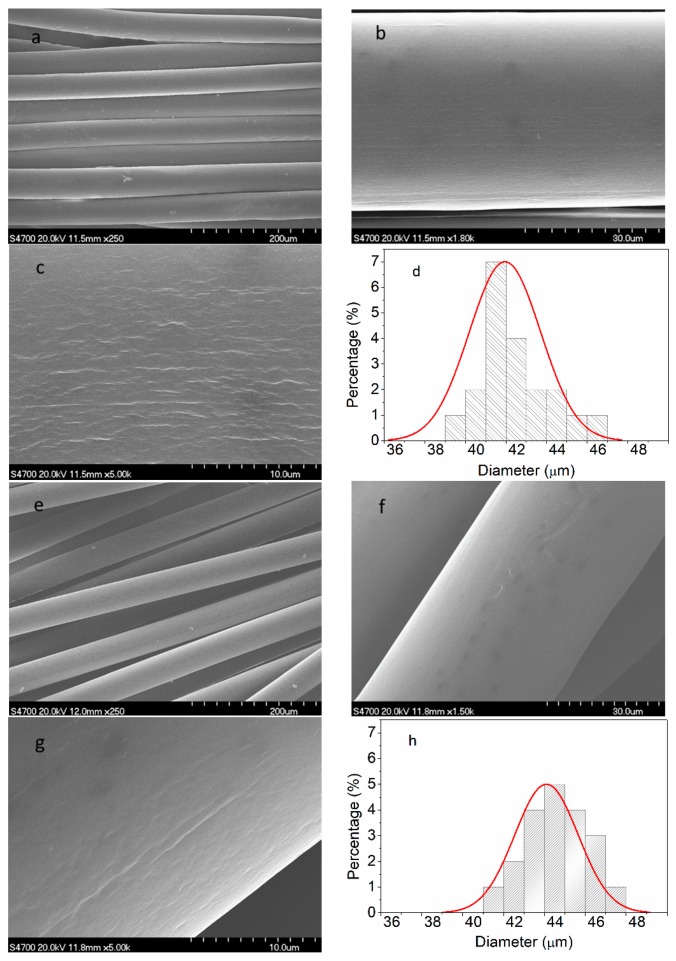
SEM images of surface morphology and corresponding diameter distribution: (**a**–**d**) the PAN nascent fibers and (**e**–**h**) the grafted MWCNT/PAN nascent composite fibers.

Figure 9 shows the surface morphology of the PAN nascent fibers and the grafted MWCNT/PAN nascent composite fibers. Compared with the PAN nascent fibers in Figure 9a,d, the grafted MWCNT/PAN nascent composite fibers in Figure 9e,h had more uniform diameter distribution than that of the PAN nascent fibers. Moreover, at the same wet spinning conditions, the average diameter of the grafted MWCNT/PAN nascent composite fibers was about 44 μm, which is larger than that of the PAN nascent fibers with a diameter of about 41 μm. These may be ascribed to the effects of the grafted MWCNT on the rheological properties of spinning solutions [33], and indicated that network structures were formed between the grafted MWCNTs and the PAN polymer during polymerization. These results are beneficial for improving the mechanical properties of the nascent composite fibers, which can be proved in Table 3. The surface morphology of the PAN nascent fibers in Figure 9b,c shows an amount of relatively parallel grooves along the axes of the fibers, which is a typical surface structure of PAN fibers by the wet spinning method. These grooves would last until the final carbon fibers, and increase the surface area and improve the interfacial interaction between the carbon fibers and polymer matrix. However, the grafted MWCNT/PAN nascent composite fibers in Figure 9f,g have a relatively smooth surface, and the grooves of which are not obvious compared to that of the PAN nascent fibers. These results indicate that the grafted MWCNT affected the phase separation and solidification behaviors and changed the parameters of crystal structure.

## 4. Conclusions

In this paper, amino-MWCNTs were obtained by chemical modification. In aqueous solvent, the PAN polymer synthesized by an AN monomer under initiator, was grafted onto the surface of the amino-MWCNTs by in situ polymerization. The grafted MWCNT/PAN nascent composite fibers with parallel grooves on the surface were prepared by the wet spinning method, which would increase the surface area of fibers and improve the interfacial interaction between the fibers and polymer matrix. The results from the FTIR spectroscopy suggest that a chemical bond was formed between the amino-MWCNTs and the PAN macromolecular chains. The shift of the G-band for amino-MWCNTs coming from Raman spectroscopy indicates that a strong interfacial interaction existed between the amino-MWCNTs and the PAN polymer. TEM images showed that the grafted MWCNTs after in situ polymerization had larger diameters, of about 20–25 nm, compared to the control amino-MWCNTs with a diameter of 10 nm. TG curves showed that the amino-MWCNTs decreased the rate of weight loss at temperatures between 300 and 470 °C, and the grafted content of the PAN polymer on the surface of amino-MWCNTs was 73.2%. The addition of the grafted MWNCTs into the PAN nascent fibers improved the degree of crystallization from 35.23 to 38.73% and the crystal size from 3.01 to 3.42 nm. The DSC data indicated that the grafted MWCNTs in the nascent composite fibers at an air atmosphere decreased the initial exothermal temperature about 5.9 °C, and broadened the range of exothermic temperature from 196.3 to 200.8 °C. The cross-sectional structure coming from SEM images showed that the PAN grafted amino-MWCNTs nascent composite fibers had more compactness structure than the PAN nascent fibers, and the surface morphology showed that the composite fibers with more uniform diameter distribution had a higher diameter than the control PAN nascent fibers. Furthermore, the incorporation of the grafted MWCNTs improved the tensile strength by 14.1% and the tensile modulus by 19.7%. These results suggest that the PAN grafted MWCNT/PAN nascent composite fibers are beneficial for preparing a high performance carbon fiber. 

## Figures and Tables

**Table 1:** XRD data for the PAN nascent fibers and the grafted MWCNT/PAN nascent composite fibers.

Samples	Degree of Crystallization (%)	Crystal Size (nm)
PAN	35.23	3.01
grafted MWCNT/PAN	38.73	3.42

**Table 2:** DSC data for the PAN nascent fibers and the grafted MWCNT/PAN nascent composite fibers in an air atmosphere.

Samples	*T*_i_ (°C)	*T*_1_ (°C)	*T*_2_ (°C)	*T*_f_ (°C)	Δ*H* (J·g^−1^)	Δ*T* (°C)	Δ*H*/Δ*T* (J·g^−1^·°C^−1^)
PAN	194.0	273.9	340.9	390.3	1546	196.3	7.9
grafted MWCNT/PAN	188.1	273.0	339.5	388.9	1470	200.8	7.3

**Table 3:** The mechanical properties of the PAN nascent fibers and the grafted MWCNT/PAN nascent composite fibers.

Samples	Tensile Strength (cN/dtex)	Tensile Modulus (cN/dtex)	Breaking Elongation (%)
PAN	3.83 ± 0.34	101.73 ± 9.32	50.63 ± 4.24
grafted MWCNT/PAN	4.37 ± 0.28	121.82 ± 8.13	32.68 ± 2.33
